# Environmental barriers matter from the early stages of functional decline among older adults in France

**DOI:** 10.1371/journal.pone.0270258

**Published:** 2022-06-22

**Authors:** Caroline Laborde, Joël Ankri, Emmanuelle Cambois

**Affiliations:** 1 Université Paris-Saclay, UVSQ, Inserm, CESP, Echappement aux anti-infectieux et pharmaco-épidémiologie, Montigny-le-Bretonneux, France; 2 Observatoire régional de santé Île-de-France, Département de l’Institut Paris Région, Paris, France; 3 Institut national d’études démographiques (Ined), Paris, France; Faculty of Health Sciences - Universidade da Beira Interior, PORTUGAL

## Abstract

**Background:**

The adaptation of living environments can preserve functional independence among older people. A few studies have suggested that this would only benefit the most impaired. But conceptual models theorize that environmental pressure gradually increases with functional decline.

**Objectives:**

We examined (1) how far different environmental barriers increased difficulties and favoured resort to assistance; (2) at what stage in functional decline environmental barriers begin to matter.

**Methods:**

We used the French cross-sectional survey CARE (2015), including 7,451 participants (60+) with at least one severe functional limitation (FL). Multinomial logistic regressions models were used to compare predicted probabilities for outdoor activities of daily living (OADL) difficulties (no OADL difficulties; difficulties but without assistance; use of assistance) among individuals with and without environmental barriers (self-reported or objective), in relation to the number of FLs.

**Results:**

Poor-quality pedestrian areas and lack of places to rest were associated with a higher probability of experiencing OADL difficulties, whatever the number of FLs; the association increased with the number of FLs. Up to 6 FLs, individuals with these barriers were more likely to report difficulties without resorting to assistance, with a decreasing association. Living in cities/towns with high diversity of food outlets was associated with a lower probability of reporting assistance, whatever the number of FLs, but with a decreasing association.

**Discussion:**

Overall, the results suggest that environmental barriers increasingly contribute to OADL difficulties with the number of FLs. Conclusions differed as to whether they tended to favour resort to assistance, but there was a clear association with food outlets, which decreased with impairment severity. The adaptation of living environments could reduce difficulties in performing activities from the early stages of decline to the most severe impairment. However, the most deteriorated functional impairments seem to generate resort to assistance whatever the quality of the environment.

## 1. Introduction

In a context of ageing populations, preserving functional independence and reducing the needs for human assistance are major policy issues. Functional independence is the ability to perform daily activities without human assistance, which involves interactions between health conditions, personal and environmental factors, as conceptualized in the disablement process [1] and the International Classification of Functioning [2]. Individuals with functional limitations can encounter difficulties shopping, getting to administrative facilities or walking around independently, due to environmental barriers between their home and shops or facilities (such as poor-quality pedestrian areas, the presence of steps, hills, or slopes). When these difficulties become insurmountable, they need to be helped and they thus lose a part of their functional independence. The *ecological theory of ageing* states that behaviours depend on the competence of the individual and the environmental pressure to which he/she is exposed, with environmental pressures increasing as an individual’s functional status decreases [3]. Therefore, with age-related functional decline, these environmental effects are particularly important for older adults [4], especially at neighbourhood level, when they become less functionally mobile [5, 6]. In recent years, adapting living environments has received growing attention from researchers [7] and policy makers [8] because it is a crucial step to preserve functional independence for older adults, [9] and more broadly their quality-of-life [10]. In this research, we examined how the presence of environmental barriers interacts with functional decline, restricting older people’s daily activities and their functional independence. We pinpoint functional situations in which improving the neighbourhood physical environment could reduce difficulties and preserve functional independence among ageing individuals. This is a major challenge to improve the wellbeing of ageing populations and to monitor their needs for assistance.

A growing body of research has shown that the neighbourhood physical environment influences older adults’ later-life restrictions. Major barriers to walking out have been highlighted, such as poor-quality pedestrian areas [11], lack of benches [12], the presence of hills or slopes in the nearby environment [13], and barriers located at the entrances to homes (such as stairs, steps between home and street, narrow doorways etc.) [14]. They increase the risk of falls [15], the apprehension when getting around outdoors [16] and they are associated with a greater risk of disability [17, 18]. In contrast, neighbourhoods with mixed land use (residential, commercial, services) [12, 19], a highly connected street network (*e*.*g*. numerous intersections) [20–22], access to green spaces [22], proximity of recreational facilities [23, 24] and food outlets [25, 26] appear to facilitate walking and decrease restrictions in later life. But the options and decisions relating to walking out and shopping could be also affected by the neighbourhood social context (for instance in terms of safety, social cohesion) [27–29], macroeconomic factors (such as area-level deprivation) [18, 30] and socioeconomic barriers (such as food prices). However, some studies have suggested that neighbourhood physical characteristics remain associated with disability, even after taking account of the socio-economic context of the neighbourhood [18, 20].

Thus the neighbourhood physical environment seems to influence disability in later life by way of two associated mechanisms [20]: physical facilitators promote social [31] and physical activity [13, 32, 33] among healthy older people and can prevent declining physical [34] and cognitive [35] functioning; physical facilitators assist older adults with functional limitations in performing daily activities and in gaining independence. Regarding the second mechanism, previous studies investigating how the neighbourhood physical environment interacts with functional status have suggested it would only benefit the most impaired, that is to say only above a given severity threshold [14, 19, 36]. Indeed, a study in the USA showed that the condition of the streets had an effect on outdoor mobility only among adults reporting severe physical impairment (whereas there was no effect among those with no physical limitations or only some) [36]. Another study in the USA found that older adults with functional limitations living in low mixed-use tracts reported more difficulties with instrumental activities, but only for those with the highest levels of functional limitations (associations were non-significant for the others) [19]. Interactions between the neighbourhood physical environment and functional capacities need to be explored in depth, investigating potential variations across the gradient of functional status.

Data from the CARE-Seniors survey of French older adults matched with geo-located data was used to investigate: (1) to what extent different environmental barriers increase difficulties and tend to favour the resort to assistance for individuals with functional limitations, (2) at which stage in functional decline environmental barriers begin to matter in terms of difficulties and resort to assistance. We hypothesized that people living in areas unsuitable for walking out and with remote services were more likely to have difficulties having outdoor activities, more likely to need assistance, and less likely to remain independent. In line with the *ecological theory of ageing*, we hypothesized that the more functional limitations there were, the more problematic environmental barriers were likely to be. We hypothesized that the neighbourhood physical environment contributed to disability all along the gradient of functional status and not only above a given severity threshold. However, we also hypothesized that the most deteriorated level of functional status would generate activity restrictions and resort to assistance whatever the quality of the environment.

## 2. Materials and methods

We used data from the French cross-sectional survey CARE-Seniors (Capacity, Aids and REsources) conducted in 2015 by the statistical department of the French Ministry of Health (Drees). CARE-Seniors was conducted on a sample of 10,628 French men and women aged 60+, representative of the population living in private homes. People with functional difficulties were oversampled, on the basis of a screening survey conducted in 2014 [37]. The participation rate in CARE-Seniors was 71%. Weights were calculated by the survey producer at individual level to take account of non-response and unequal probabilities of sampling (by gender, age, and functional status). The survey adopted the International classification of Functioning [1, 2, 38, 39] as a conceptual framework. The survey used face-to-face interviews to collect individual data; contextual datasets including the geo-location of shops, healthcare facilities and services in the city were matched *a posteriori* using the respondents’ city/town of residence. The CARE Seniors survey was compulsory, recognised as being in the public interest, and approved for its statistical quality by the CNIS (National council for statistical information). All participants were informed before data collection and, in accordance with the General Data Protection Regulation (GDPR), they have a right of access and rectification.

### 2.1. Restrictions in outdoor activities of daily living (OADL)

On the basis of the elementary (ADL) and instrumental (IADL) activities of daily living [40, 41], the respondents were asked, for each of them: "do you have difficulty in performing *activity* on your own (even when using your usual aid devices)", with the following response categories: "No", "Yes some difficulty", "Yes a lot of difficulty", "Yes, and I cannot do it alone". Respondents who answered positively were further questioned as to whether they received assistance from someone else. Among all the IADLs assessed in the CARE-Seniors survey, we selected a set of four "outdoor" activities of daily living (OADL) with potential links with the outdoor environment: shopping, getting out of the house, using public transport, carrying out administrative procedures (performing the latter activity can be conditioned by access to the post office or the bank—especially for those aged 70 or over, half of whom do not use the Internet in France [42]). Because a part of the older population frequently does not take on the responsibility for shopping or carry out administrative procedures in daily life [43], individuals who reported not doing these activities were asked if it was "mainly because of their health status or their age”; if the response was no, we classified them as having no difficulty.

For each activity, we constructed a measure classifying participants into three groups (S1 Table): no difficulties; difficulties but no resort to assistance; resort to assistance. As with previous studies [18, 44–46], we created a summary measure combining answers for each outdoor activity and retaining the lowest performance. We built a 3-level indicator to identify participants who resorted to help or had difficulties (with assistive devices if used): no OADL difficulties, difficulties but no resort to assistance, resort to assistance. These three levels reflect a severity gradient under the hypothesis that individuals reporting assistance were more severely restricted than those who did not, even if they had difficulty performing the activity. Making a distinction between experiencing difficulties and receiving assistance enabled us to examine the issue of independence in outdoor activities more fully. We were aware of possible misclassifications. The category "difficulties but no resort to assistance" could comprise individuals who underestimated the assistance received because it seemed natural or unimportant to them [47] and people whose need for assistance had not been met [48, 49]. The category “resort to assistance” could also include individuals who were assisted but who did not necessarily need it. We will discuss our results in the light of these limitations.

This indicator is based on activity restrictions that individuals encounter using their usual aid devices, which could compensate for functional limitations and reduce the risk of activity restrictions [50, 51]. In this sense, this indicator reflects the actual difficulties experienced by individuals deploying their (usual) maximum capacities.

### 2.2 Functional limitations (FLs)

The questions on self-reported functional limitations (FLs) were based on Nagi’s measures [38]. Respondents were asked whether they could perform basic tasks such as climbing stairs, hearing a conversation (including when using usual aids) and whether they would answer "yes with no difficulty", "yes, with some difficulty", "yes, with a lot of difficulty" or "not able at all". We considered nineteen motor, sensory, and cognitive limitations (S2 Table). In this study, we considered severe FLs corresponding to the answers "A lot of difficulty” or “Not able at all". This selection enabled us to focus on the most critical situations. We used the number of limitations among the nineteen items, up to a threshold of 10+, which amounted to 5% of the study population.

### 2.3. Environmental barriers

To assess the neighbourhood physical environment, we used three different measures of environmental barriers, mobilizing either self-report or objective data. This approach enabled us to observe whether the results varied or were stable depending on the measure and on the collection method.

#### Low diversity of facilities in the city of residence

This environmental characteristic was explored using a contextual dataset produced by the national statistics institute (INSEE) and matched with the CARE-Seniors dataset. It provides a variable on the driving distance between the city centre (town hall) and various facilities. We did not have any information on the driving or walking distance from the individual homes. Indeed, there are certainly inequalities in access to resources within a city [52, 53]. However, this variable enabled us to approach this information. We selected 16 facilities classified into general facilities (police, bank, post office, and hairdresser), food outlets (grocery stores, bakeries, restaurants, and supermarkets) and health facilities (general practitioner, cardiologist, dentist, nurse, physiotherapist, pharmacy, medical laboratory, and optician). INSEE considered the driving distance to be zero if a facility was available within the city. For each category of facility, we divided the sample into those living in cities with all food outlets, general facilities, and health facilities (high diversity) and those with few/sparse facilities (low diversity). It should be noted that we did not have any information on how rural or urban the environment was nor about the size of the town or city. These variables are nevertheless certainly related to access to shops and services [54].

#### Barriers in the near environment

In the survey questionnaire, respondents were asked about barriers when out walking with the following question: "When you are walking or using your wheelchair, are you bothered by … (1) *poor-quality pedestrian areas (crowded/busy pavements*, *absence of pavements*, *etc*.*);* (2) *the steepness of a hill or slope;* (3) *the lack of places to have a rest (benches etc*.*);* (4) *the absence of*, *or difficult access to public toilets"*. Four dummy variables were constructed on the basis of these categories: poor-quality pedestrian areas; presence of hills/slopes; lack of places to rest; no easy access to public toilets. In contrast with the previous variables, these measures could be influenced by the individual’s functional capacities and perceptions, as they were self-reported. However, Portegijs [14] showed that older adults for whom barriers were objectively recorded were more likely to perceive and report barriers, regardless of their functional status.

#### Barriers in the immediate environment

Respondents were asked whether they had to use stairs or steps to get out of their home. A dummy variable “presence of stairs/steps” was constructed, corresponding to a barrier for getting out of the home. In contrast with the previous variable, respondents were not asked to link this situation to possible mobility difficulties, but only report it as being there or not.

### 2.4. Covariates

#### Socio-demographic covariates

We considered gender, age (as a continuous variable) and education as covariates. Age was the strongest factor associated with both difficulties in daily activities [55] and perceptions of the barriers [56]. Persistent gender and social inequalities in disability have been found in the international literature: women and people with lower educational status have a greater likelihood of having disabilities [57–60]. Educational level was measured according to the International Standard Classification of Education (ISCED 2011): (0) *no diploma* (ISCED 0), (1) *low educational level* (ISCED 1–2), (2) *moderate educational level* (ISCED 3) and (3) *high educational level* (ISCED 4–8) [61].

#### Living arrangements and social network

To reflect possible variations in the assistance received according to the support available [62, 63], we built a dummy variable "living alone", and a variable on the frequency of relationships and contacts with family members, friends, and neighbours in the past 12 months: *often* (at least several times a month), *rarely* (at least once a year), and *never* (never in the past 12 months or has no family, friends, or neighbours).

#### Physical and mental health status

Diseases can have a direct impact on performing outdoor activities [64, 65] and can exacerbate sensitivity to the neighbourhood physical environment [66–69]; in the model, we included the (self-reported) number of diagnosed chronic diseases in the past 12 months and the five-item Mental Health Inventory score (MHI-5) (below 56) [70, 71].

### 2.5. Statistical analysis

We first analysed the characteristics of the study sample using chi-square tests to compare categorical variables and adjusted Wald tests to compare continuous variables. Then we used multinomial logistic regression models to examine associations between the 3-level variable indicating whether an individual had no OADL difficulties, difficulties but no resort to assistance, resort to assistance (dependent variable) and environmental barriers (independent variables). These analyses were multivariate and adjusted on variables significant at P<0.05 in univariate analysis (number of FLs, socio-demographic covariates, living arrangements and social network, physical and mental health status). We ran separate models for each environmental barrier, and we ran full models on all the environmental barriers simultaneously (only the full models are presented in this paper).

Two multinomial logistic regression models are presented: (1) full adjusted model, to compare probabilities for individuals living with and without barriers; (2) model 1 broken down into the number of FLs from 1 to 10+, to test the hypothesis of changes in associations between the environment and OADL restrictions with the severity of the functional status. Using these two models, we estimated predicted probabilities, i.e. Average Adjusted Predictions (AAPs) in model 1 and Adjusted Predictions at Representative values (APRs) in model 2, which are easier to interpret than relative risk ratios (RRRs) [72]. The results are shown in the form of predictive probabilities with 95% confidence intervals (CIs). To estimate the difference between predicted probabilities, we produced Average Marginal Effects (AMEs) in model 1 and Marginal Effects at Representative Values (MERVs) in model 2. Statistical significance was established at p<0.10. We did not perform separate analyses for people using a wheelchair because they were a minority in our sample (2%). Sampling weights were applied to all analyses to take account of the CARE-Seniors study design. Statistical analyses were conducted on STATA 16.1.

## 3. Results

Our sample comprised 7,451 respondents who reported at least one severe FL (out of a total of 10,628). In our sample, the average age was 76.2 (SD 0.2), and the majority were women. Most of the sample had a low educational level or no diploma (Table 1). Most were living with someone and often saw their families, friends, and neighbours. Almost 40% of our sample were living in a city with low diversity of food outlets (among them, 20% did not have a supermarket in their city, 37% no grocery store and 43% had neither a supermarket nor a grocery store). Less than 20% reported barriers in their near environment and 55% reported having to use stairs to get out.

**Table 1:** General characteristics of the study sample. CARE-Seniors Ménages Survey (60+ with at least 1 FL), 2015, France.

	Total	No OADL difficulties	Difficulties but no resort to assistance	Resort to assistance	*p-value* *
**Total, n(%)**	7,451 (100.0%)	1,964 (52.5%)	966 (11.6%)	4,521 (35.9%)	
**Low diversity of facilities in the city/town of residence**					
General, n(%)^a^	3,412 (47.5%)	965 (52.0%)	412 (39.0%)	2,035 (43.6%)	*<0*.*001*
Food, n(%)^a^	2,894 (39.5%)	842 (44.2%)	321 (28.6%)	1,731 (36.2%)	*<0*.*001*
Health, n(%)^a^	4,214 (57.3%)	1,184 (61.5%)	505 (49.2%)	2,525 (53.8%)	*<0*.*001*
**Physical barriers in the immediate environment**					
Stairs/Steps to get out, n(%)^a^	3,714 (54.6%)	1,048 (51.8%)	512 (59.3%)	2,154 (57.8%)	*0*.*004*
**Physical barriers in the near environment **					
Poor-quality pedestrian areas, n(%)^a^	1,600 (17.2%)	287 (11.9%)	252 (26.6%)	1,061 (22.0%)	*<0*.*001*
Presence of hills/slopes, n(%)^a^	1,502 (17.1%)	303 (12.6%)	243 (25.6%)	956 (21.1%)	*<0*.*001*
Lack of places to rest, n(%)^a^	1,362 (15.6%)	251 (10.6%)	231 (24.7%)	880 (20.1%)	*<0*.*001*
No easy access to toilets, n(%)^a^	849 (12.2%)	249 (11.7%)	123 (14.4%)	477 (12.1%)	*<0*.*001*
**Number of severe functional limitations (FLs)**					
Mean (SD)^b^	3.2 (0.04)	1.8 (0.04)	3.6 (0.11)	5.1 (0.07)	*<0*.*001*
**Gender**, n(%)^a^					
Men	2,546 (38.4%)	893 (45.8%)	345 (34.6%)	1,308 (28.9%)	*<0*.*001*
Women	4,905 (61.6%)	1,071 (54.2%)	621 (65.4%)	3,213 (71.1%)	
**Age—**Mean (SD)^b^	76.2(0.20)	72.5(0.26)	77.5(0.47)	81.0(0.26)	*<0*.*001*
**Level of education**, n(%)^a^					
No diploma	2,268 (25.6%)	431 (20.2%)	255 (24.9%)	1,582 (33.7%)	*<0*.*001*
Low educational level	2,936 (37.7%)	709 (33.6%)	376 (43.1%)	1,851 (41.8%)	
Medium educational level	1,736 (27.6%)	628 (34.6%)	247 (22.9%)	861 (19.0%)	
High educational level	511 (9.1%)	196 (11.6%)	88 (9.0%)	227 (5.4%)	
**Living alone**, n(%)^a^					
Yes	3,590 (40.3%)	712 (32.4%)	505 (50.5%)	2,373 (48.7%)	*<0*.*001*
No	3,861 (59.7%)	1,252 (67.7%)	461 (49.5%)	2,148 (51.3%)	
**Frequency of relationships with family members (other than co-residents)**, n(%)^a^					
Often	5,376 (71.5%)	1,387 (70.6%)	615 (64.8%)	3,374 (74.9%)	*<0*.*001*
Rarely	1,675 (24.6%)	503 (26.9%)	284 (29.8%)	888 (19.5%)	
Never	383 (4.0%)	72 (2.5%)	65 (5.4%)	246 (5.7%)	
**Frequency of relationships with friends and neighbours**, n(%)^a^					
Often	4,445 (65.8%)	1,381 (73.2%)	592 (60.2%)	2,472 (57.0%)	*<0*.*001*
Rarely	1,506 (18.6%)	360 (17.8%)	178 (17.7%)	968 (19.9%)	
Never	1,466 (15.6%)	214 (9.0%)	192 (22.2%)	1,060 (23.2%)	
**Number of diagnosed chronic diseases**					
Mean (SD)^b^	3.2 (0.04)	2.6 (0.06)	3.5 (0.12)	3.8 (0.06)	*<0*.*001*
**Psychological distress**, n(%)^a^					
Yes	3,008 (31.0%)	573 (21.9%)	491 (46.0%)	1,944 (39.5%)	*<0*.*001*
No	4,443 (69.0%)	1,391 (78.1%)	475 (54.0%)	2,577 (60.5%)	

Percentages and Means (SD) are weighted

* *P-values* are derived from

^a^ chi-square tests or

^b^ adjusted Wald tests.

Half of them did not report any OADL difficulties (53%); 12% reported difficulties but without resort to assistance; 36% reported resort to assistance. The mean number of severe FLs increased across these three categories, confirming the assumption of a severity gradient. Those with OADL difficulties were more likely to be living in cities with high diversity of facilities, but they were also more likely to report barriers in their immediate or near vicinity. Those having difficulties but reporting no assistance were more likely to report poor-quality pedestrian areas, a lack of places to rest and the presence of hills/slopes compared to those reporting assistance.

### 3.1. OADL difficulties and environmental barriers

Adjusted multivariate multinomial logistic regression models (Model 1) showed that living in an environment with barriers was associated with a higher predicted probability of having OADL difficulties (except for general and health facilities) and of resorting to assistance (Table 2). Differences were statistically significant for 4 out of the 8 barriers tested. Individuals living in cities with low diversity of food outlets tended to resort more to assistance than their counterparts living in cities with high diversity of food outlets (AAP = 35.8%, 95% CI [32.7, 39.0] for low diversity of food outlets; AAP = 31.8%, 95% CI [29.8, 33.8] for high diversity of food outlets, p-value = 0.056). Regarding the near environment, poor-quality pedestrian areas, and lack of places to rest increased the predicted probability of difficulties, particularly without resort to assistance. Regarding the immediate environment, having to use stairs/steps to get out increased the likelihood of having OADL restrictions (AAP of having no OADL difficulties = 53.6%, 95% CI [51.6, 55.6] for those with stairs/steps; AAP of having no OADL difficulties = 57.1%, 95% CI [54.7, 59.6] for those without stairs/steps, p-value = 0.026).

**Table 2:** Average Adjusted Predictions (AAPs)° of OADL difficulties and resort to assistance (Model 1). CARE-Seniors Ménages Survey (60+ with at least 1 FL), 2015, France.

	No OADL difficulties (n = 1,964)			Difficulties but no resort to assistance (n = 966)					Resort to assistance (n = 4,521)				
	AAPs°	95% CI	*p-value* *	AAPs°	95% CI			*p-value* *	AAPs°	95% CI			*p-value* *
**Diversity of facilities in the city/town of residence**													
Food High	55.8%	53.5, 58.2	*0*.*452*	12.3%	10.7, 14.0			*0*.*191*	31.8%	29.8, 33.8			***0*.*056***
Low	54.0%	50.8, 57.3		10.2%	8.0, 12.4				35.8%	32.7, 39.0			
General High	54.9%	52.4, 57.3	*0*.*788*	11.9%	10.0, 13.9			*0*.*708*	33.2%	31.0, 35.4			*0*.*959*
Low	55.5%	52.6, 58,3		11.2%	8.9, 13.6				33.3%	30.6, 36.0			
Health High	53.8%	50.9, 56.7	*0*.*261*	11.9%	9.8, 14.0			*0*.*773*	34.3%	31.6, 37.1			*0*.*326*
Low	56.2%	53.8, 58.6		11.4%	9.5, 13.4				32.4%	30.2, 34.5			
**Physical barriers in the immediate environment**													
Stairs/steps No	57.1%	54.7, 59.6	***0*.*026***	11.1%	9.4, 12.7			*0*.*375*	31.8%	29.5, 34.1			***0*.*097***
Yes	53.6%	51.6, 55.6		12.1%	10.5, 13.7				34.3%	32.5, 36.2			
**Physical barriers in the near environment**													
Poor-quality pedestrian areas													
No	56.3%	54.6, 58.1	***0*.*003***	10.9%	9.6, 12.1			***0*.*012***	32.8%	31.1, 34.4			*0*.*167*
Yes	49.5%	45.2, 53.8		14.9%	11.8, 18.0				35.6%	31.7, 39.5			
Presence of hills and slopes													
No	55.6%	53.9, 57.4	*0*.*239*	11.2%	9.9, 12.5			*0*.*172*	33.2%	31.5, 34.9			*0*.*763*
Yes	53.0%	49.1, 56.9		13.3%	10.5, 16.0				33.7%	30.3, 37.2			
Lack of places to rest													
No	56.2%	54.5, 58.0	***0*.*006***	11.0%	9.7, 12.3			***0*.*019***	32.8%	31.1, 34.5			*0*.*169*
Yes	49.5%	45.1, 53.9		14.8%	11.7, 17.9				35.7%	31.8, 39.6			
No easy access to public toilets													
No	54.8%	53.2, 56.5	*0*.*392*	11.9%	10.6, 13.1			*0*.*427*	33.3%	31.8, 34.8			*0*.*750*
Yes	57.1%	52.2, 62.1		10.5%	7.4, 13.5				32.4%	27.4, 37.4			

° Adjusted on gender, age as a continuous variable, level of education, living alone or not, frequency of relationships with family members (other than co-residents), with friends and neighbours, number of diagnosed chronic diseases, psychological distress, number of severe functional limitations and all the environmental barriers.

* *p-values* were used to test whether the differences (AMEs) between the predicted probabilities (AAPs) were significantly different from 0.

### 3.2. OADL difficulties, environmental barriers, and the number of FLs

**Fig 1:**
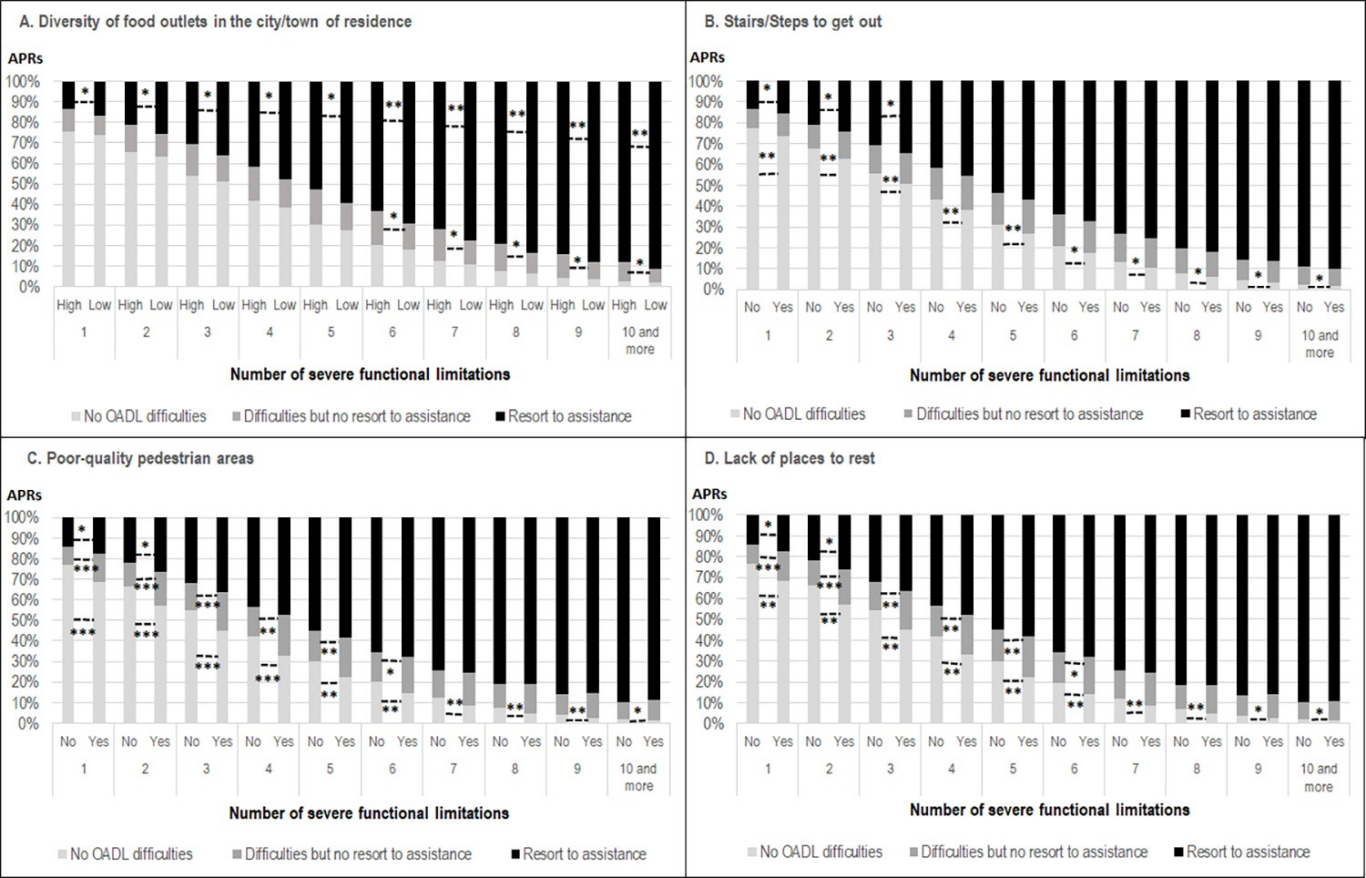
Adjusted Predictions at Representative values° (APRs) of OADL difficulties and resort to assistance, from 1 to 10+ severe FLs (Model 2): (A) high vs low diversity of food outlets in the city/town of residence; (B) with/without stairs/steps to get out; (C) with/without poor-quality pedestrian areas; (D) with/without places to have a rest. CARE-Seniors Ménages Survey (60+ with at least 1 FL) 2015, France. *p<0,10; ** p<0,05; *** p<0,01. °Adjusted on gender, age as a continuous variable, level of education, living alone, frequency of relationships with family members (other than co-residents), with friends and neighbours, number of diagnosed chronic diseases, psychological distress, number of severe functional limitations and all the environmental barriers. * *p-values* were used to test whether the differences (MERVs) between the predicted probabilities (APRs) were significantly different from 0.

To address the question of a gradient in the association according to the number of FLs, Adjusted Predictions at Representative values (APRs) of OADL restrictions were estimated for the 4 barriers that showed a significant association in the previous model. Fig 1 highlights a dose-response association of OADL difficulties with the number of FLs: the probability of having no OADL difficulties strongly declined and the resort to assistance increased greatly with the number of FLs. We also found an interaction between environmental barriers and the number of FLs. Table 3 indicates the relative predictive probabilities (RAPRs) of OADL difficulties, dividing the APRs of those who reported no barriers by the APRs of those who did report barriers (APRs are indicated in Table 3 and illustrated in Fig 1).

**Table 3:** Relative Adjusted Predictions at Representative values ° (APRs) of OADL difficulties and resort to assistance, for 1 to 10+ severe FLs (Calculations based on APRs estimated in model 2). CARE-Seniors Ménages Survey (60+ with at least 1 FL) 2015, France.

	No OADL difficulties (n = 1,964)				Difficulties but no resort to assistance (n = 966)				Resort to assistance (n = 4,521)			
Number of severe FLs	No barrier APR°(95% CI)	Barrier APR°(95% CI)	*p-value* *	RAPR^a^	No barrier APR°(95% CI)	Barrier APR°(95% CI)	*p-value* *	RAPR^a^	No barrier APR°(95% CI)	Barrier APR°(95% CI)	*p-value* *	RAPR^a^
	**Barrier = Low diversity of food outlets in the city/ town of residence**											
1	0.76(0.73,0.79)	0.74(0.7,0.79)	*0*.*548*	1.02	0.11(0.08,0.13)	0.09(0.07,0.12)	*0*.*384*	1.15	0.14(0.11,0.16)	0.17(0.13,0.2)	***0*.*095***	0.82
2	0.66(0.63,0.69)	0.63(0.59,0.68)	*0*.*476*	1.04	0.13(0.11,0.15)	0.11(0.09,0.14)	*0*.*299*	1.17	0.21(0.19,0.24)	0.25(0.21,0.29)	***0*.*081***	0.83
3	0.54(0.51,0.57)	0.51(0.47,0.56)	*0*.*416*	1.06	0.15(0.13,0.17)	0.13(0.1,0.16)	*0*.*218*	1.20	0.31(0.28,0.34)	0.36(0.32,0.4)	***0*.*068***	0.85
4	0.42(0.38,0.46)	0.39(0.34,0.43)	*0*.*367*	1.08	0.17(0.14,0.19)	0.14(0.11,0.17)	*0*.*154*	1.22	0.42(0.38,0.45)	0.48(0.43,0.52)	***0*.*056***	0.87
5	0.3(0.25,0.35)	0.27(0.22,0.32)	*0*.*327*	1.10	0.17(0.15,0.2)	0.14(0.11,0.17)	*0*.*110*	1.25	0.53(0.49,0.57)	0.59(0.54,0.64)	***0*.*047***	0.89
6	0.2(0.15,0.25)	0.18(0.13,0.22)	*0*.*296*	1.13	0.17(0.14,0.19)	0.13(0.1,0.16)	***0*.*083***	1.28	0.63(0.59,0.68)	0.69(0.64,0.74)	***0*.*040***	0.91
7	0.13(0.09,0.17)	0.11(0.07,0.14)	*0*.*273*	1.16	0.15(0.12,0.18)	0.12(0.08,0.15)	***0*.*069***	1.31	0.72(0.67,0.77)	0.78(0.73,0.82)	***0*.*036***	0.93
8	0.07(0.04,0.1)	0.06(0.04,0.09)	*0*.*258*	1.19	0.13(0.1,0.17)	0.1(0.07,0.13)	***0*.*062***	1.33	0.79(0.75,0.84)	0.84(0.8,0.88)	***0*.*036***	0.95
9	0.04(0.02,0.06)	0.03(0.02,0.05)	*0*.*250*	1.21	0.11(0.08,0.15)	0.08(0.05,0.11)	***0*.*060***	1.35	0.85(0.8,0.89)	0.88(0.84,0.92)	***0*.*039***	0.96
10 +	0.02(0.01,0.04)	0.02(0.01,0.03)	*0*.*247*	1.23	0.09(0.06,0.13)	0.07(0.04,0.1)	***0*.*061***	1.37	0.88(0.85,0.92)	0.91(0.88,0.94)	***0*.*045***	0.97
	**Barrier = Stairs/steps to get out**											
1	0.77(0.74,0.81)	0.73(0.7,0.77)	***0*.*027***	1.05	0.09(0.07,0.11)	0.11(0.09,0.13)	*0*.*172*	0.86	0.13(0.11,0.16)	0.16(0.13,0.18)	***0*.*054***	0.84
2	0.67(0.64,0.71)	0.63(0.6,0.66)	***0*.*026***	1.08	0.12(0.1,0.14)	0.13(0.11,0.15)	*0*.*229*	0.88	0.21(0.18,0.24)	0.24(0.22,0.27)	***0*.*062***	0.87
3	0.56(0.52,0.59)	0.51(0.48,0.53)	***0*.*026***	1.10	0.14(0.12,0.16)	0.15(0.13,0.17)	*0*.*325*	0.91	0.31(0.27,0.34)	0.34(0.32,0.37)	***0*.*075***	0.89
4	0.43(0.39,0.47)	0.38(0.35,0.42)	***0*.*027***	1.13	0.15(0.13,0.17)	0.16(0.14,0.18)	*0*.*462*	0.93	0.42(0.38,0.46)	0.46(0.42,0.49)	*0*.*103*	0.91
5	0.31(0.26,0.36)	0.27(0.23,0.31)	***0*.*028***	1.17	0.15(0.13,0.18)	0.16(0.14,0.19)	*0*.*627*	0.95	0.53(0.49,0.58)	0.57(0.53,0.61)	*0*.*124*	0.94
6	0.21(0.16,0.25)	0.17(0.13,0.21)	***0*.*031***	1.20	0.15(0.12,0.18)	0.15(0.13,0.18)	*0*.*789*	0.97	0.64(0.59,0.69)	0.67(0.63,0.72)	*0*.*171*	0.95
7	0.13(0.09,0.17)	0.11(0.07,0.14)	***0*.*037***	1.23	0.14(0.11,0.17)	0.14(0.11,0.17)	*0*.*920*	0.99	0.73(0.68,0.78)	0.76(0.71,0.8)	*0*.*243*	0.97
8	0.08(0.05,0.11)	0.06(0.04,0.09)	***0*.*046***	1.25	0.12(0.09,0.15)	0.12(0.09,0.15)	*0*.*988*	1.00	0.8(0.76,0.85)	0.82(0.78,0.86)	*0*.*340*	0.98
9	0.04(0.02,0.06)	0.03(0.02,0.05)	***0*.*057***	1.27	0.1(0.07,0.13)	0.1(0.07,0.13)	*0*.*931*	1.01	0.86(0.82,0.9)	0.87(0.83,0.9)	*0*.*452*	0.99
10 +	0.02(0.01,0.04)	0.02(0.01,0.03)	***0*.*070***	1.28	0.09(0.05,0.12)	0.08(0.05,0.11)	*0*.*898*	1.01	0.89(0.86,0.93)	0.9(0.86,0.93)	*0*.*561*	0.99
	**Barrier = Poor-quality pedestrian areas**											
1	0.77(0.74,0.79)	0.69(0.63,0.74)	***0*.*003***	1.12	0.09(0.08,0.11)	0.14(0.1,0.17)	***0*.*004***	0.67	0.14(0.12,0.16)	0.18(0.13,0.22)	***0*.*060***	0.80
2	0.67(0.64,0.69)	0.57(0.51,0.63)	***0*.*003***	1.16	0.12(0.1,0.13)	0.17(0.13,0.2)	***0*.*005***	0.70	0.22(0.2,0.24)	0.26(0.21,0.31)	***0*.*0*** *77*	0.84
3	0.55(0.52,0.57)	0.45(0.39,0.51)	***0*.*004***	1.21	0.13(0.12,0.15)	0.19(0.15,0.22)	***0*.*009***	0.73	0.32(0.3,0.34)	0.36(0.31,0.42)	*0*.*106*	0.88
4	0.42(0.39,0.45)	0.33(0.27,0.39)	***0*.*004***	1.27	0.15(0.13,0.17)	0.19(0.16,0.23)	***0*.*019***	0.76	0.43(0.4,0.46)	0.48(0.42,0.53)	*0*.*157*	0.91
5	0.3(0.26,0.34)	0.23(0.17,0.28)	***0*.*006***	1.33	0.15(0.13,0.17)	0.19(0.15,0.23)	***0*.*042***	0.79	0.55(0.51,0.59)	0.58(0.52,0.64)	*0*.*243*	0.94
6	0.2(0.16,0.24)	0.14(0.1,0.19)	***0*.*009***	1.38	0.15(0.12,0.17)	0.18(0.14,0.22)	***0*.*082***	0.81	0.66(0.61,0.7)	0.68(0.62,0.74)	*0*.*388*	0.97
7	0.12(0.09,0.16)	0.09(0.05,0.12)	***0*.*014***	1.43	0.13(0.11,0.16)	0.16(0.12,0.2)	*0*.*134*	0.82	0.74(0.7,0.79)	0.75(0.7,0.81)	*0*.*604*	0.99
8	0.07(0.04,0.1)	0.05(0.03,0.07)	***0*.*022***	1.47	0.12(0.09,0.14)	0.14(0.1,0.18)	*0*.*183*	0.84	0.81(0.77,0.85)	0.81(0.76,0.86)	*0*.*872*	1.00
9	0.04(0.02,0.06)	0.03(0.01,0.04)	***0*.*034***	1.49	0.1(0.07,0.13)	0.12(0.08,0.16)	*0*.*222*	0.84	0.86(0.82,0.9)	0.86(0.81,0.9)	*0*.*863*	1.01
10 +	0.02(0.01,0.03)	0.01(0.01,0.02)	***0*.*049***	1.50	0.08(0.05,0.11)	0.1(0.06,0.14)	*0*.*247*	0.84	0.9(0.86,0.93)	0.89(0.85,0.93)	*0*.*653*	1.01
	**Barrier = Lack of places to rest**											
1	0.77(0.74,0.79)	0.69(0.63,0.74)	***0*.*005***	1.12	0.09(0.08,0.11)	0.14(0.1,0.17)	***0*.*006***	0.68	0.14(0.12,0.16)	0.18(0.13,0.22)	***0*.*068***	0.80
2	0.66(0.64,0.69)	0.57(0.51,0.63)	***0*.*005***	1.16	0.12(0.1,0.13)	0.16(0.13,0.2)	***0*.*008***	0.71	0.22(0.2,0.24)	0.26(0.21,0.31)	***0*.*084***	0.84
3	0.54(0.52,0.57)	0.45(0.39,0.51)	***0*.*006***	1.21	0.14(0.12,0.15)	0.18(0.15,0.22)	***0*.*014***	0.74	0.32(0.29,0.34)	0.37(0.31,0.42)	*0*.*113*	0.87
4	0.42(0.38,0.45)	0.33(0.27,0.39)	***0*.*008***	1.26	0.15(0.13,0.17)	0.19(0.15,0.23)	***0*.*029***	0.77	0.43(0.4,0.46)	0.48(0.42,0.54)	*0*.*161*	0.91
5	0.3(0.26,0.34)	0.23(0.17,0.28)	***0*.*010***	1.32	0.15(0.13,0.17)	0.19(0.15,0.23)	***0*.*060***	0.80	0.55(0.51,0.59)	0.58(0.53,0.64)	*0*.*240*	0.94
6	0.2(0.16,0.24)	0.14(0.1,0.19)	***0*.*014***	1.38	0.15(0.12,0.17)	0.18(0.14,0.22)	***0*.*097***	0.82	0.66(0.61,0.7)	0.68(0.62,0.74)	*0*.*371*	0.97
7	0.12(0.09,0.16)	0.09(0.05,0.12)	***0*.*020***	1.43	0.13(0.11,0.16)	0.16(0.12,0.2)	*0*.*174*	0.84	0.74(0.7,0.79)	0.76(0.7,0.81)	*0*.*565*	0.99
8	0.07(0.04,0.1)	0.05(0.03,0.07)	***0*.*029***	1.46	0.12(0.09,0.15)	0.14(0.1,0.18)	*0*.*232*	0.85	0.81(0.77,0.85)	0.81(0.76,0.86)	*0*.*809*	1.00
9	0.04(0.02,0.06)	0.03(0.01,0.04)	***0*.*043***	1.48	0.1(0.07,0.13)	0.12(0.07,0.16)	*0*.*276*	0.86	0.86(0.82,0.9)	0.86(0.81,0.9)	*0*.*944*	1.00
10 +	0.02(0.01,0.03)	0.01(0.01,0.02)	***0*.*058***	1.50	0.08(0.05,0.11)	0.1(0.06,0.13)	*0*.*305*	0.86	0.9(0.86,0.93)	0.89(0.85,0.93)	*0*.*738*	1.01

° Adjusted on gender, age as a continuous variable, level of education, living alone, frequency of relationships with family members (other than co-residents), with friends and neighbours, number of diagnosed chronic diseases, psychological distress, number of severe functional limitations and all the environmental barriers.

^a^ We calculated a Relative Adjusted Predictions at Representative values: RAPR = APR (no barrier) / APR (barrier)

* p-values were used to test whether the differences (MERVs) between the predicted probabilities (APRs) were significantly different from 0.

Fig 1A shows that living in cities with low diversity of food outlets increased the probability of reporting resort to assistance at an early stage in functional decline and up to 10+ FLs. Above 6 FLs, individuals with access to higher diversity of food outlets reported fewer OADL difficulties (not significant) and more difficulties without resort to assistance (p<0.10), than those with lower diversity of food outlets. Table 3 indicates that the excess probability of using assistance for those with lower diversity of food outlets in their city decreased gradually with the number of severe FLs (for 1 FL, RAPR = 14%/17% = 0.82; for 10 FLs, RAPR = 88%/91% = 0.97). The excess probability of difficulties with no resort to assistance for those with higher diversity of food outlets in their city increased gradually.

Fig 1B shows that the individuals having to use stairs/steps to get out were more likely to have restrictions than those not reporting the use of stairs/steps, whatever the number of FLs; the excess probability of restrictions increased with the number of FLs. They tended to report more resort to assistance, but only when they had 1–3 FLs; above 3 FLs, the probability of being assisted was still higher but not significantly.

Fig 1C and 1D show that individuals living in poor-quality pedestrian areas and with a lack of places to rest were more likely to have OADL restrictions than those not reporting these environmental barriers, whatever the number of FLs; the excess probability of restrictions substantially increased with the number of FLs (Table 3). Up to 7 FLs, individuals living with these barriers were more likely to report difficulties without resorting to assistance than individuals who did not report these barriers; for 7+ FLs, the difference was no longer significant.

## 4. Discussion

In this study, we analysed the associations between environmental barriers and OADL restrictions among older adults with one or more severe FLs. We found that the more functional limitations there were, the lower was the probability of having no OADL restrictions, and the higher was the probability of reporting help. Our results also provided further evidence that cities with low diversity of food outlets, poor-quality pedestrian areas, and a lack of places to rest were a challenge for older individuals’ outdoor activities. One major contribution of our study is that environmental barriers interact with the gradient of functional status thus contributing to difficulties in OADL from the early stages of functional decline. This result is consistent with the conceptual models of disablement [1, 2] and the *ecological theory of ageing* [3].

Regarding the availability of facilities in the city, our results showed an association between low diversity of food outlets and OADL difficulties (although not statistically significant), as was shown in other studies for shopping restrictions [25, 26, 73]. In addition, individuals having low diversity of food outlets were more likely to have difficulties and receive assistance, even at an early stage in their functional decline and up to the most deteriorated status; but the strength of the association tended to decrease with the number of FLs. Unlike other studies [14, 19, 36], we did not find that barriers became a concern only above a certain severity threshold in functional status. Cities with high diversity of food outlets seemed to preserve the individuals’ independence all along the gradient of functional status. Another result suggests an associated mechanism through which the neighbourhood physical environment contributes to functional independence: the most impaired individuals (6+ severe FLs) having high diversity of food outlets had a more marked tendency to experience difficulties, but without resort to assistance. This result is congruent with Brenner and Clarke, who found that areas favouring walking were associated with greater difficulties in activities for women [18]. The authors hypothesized that women living in areas conducive to walking were more likely to travel on foot and therefore be exposed to barriers. Cities with high diversity of food outlets could be an incentive for individuals to keep on shopping independently, even if it becomes difficult. However, as mentioned earlier, our category "difficulty but no resort to assistance" could include unmet needs for assistance. Another explanation would be that well-to-do neighbourhoods with food outlets could attract people who tend to age in place and experience a growing number of functional problems developing over the years. From an intervention standpoint, our results suggest that improving access to food outlets in a neighbourhood could be a step towards preserving independence and reducing the need for assistance for all older people whatever their functional status.

In our study, as in Etman’s study [74], general and health facilities were not associated with OADL restrictions. A first hypothesis is that the general and health facilities selected in this study structure older adults’ living space less strongly than the food-related facilities. A second hypothesis is the presence of two opposite associations: on the one hand, cities with general and health facilities provide a protective setting for older people; on the other, cities where many older residents require attention provide better access to health and general services. From a methodological point of view, it is worth noting that in our sample 57% did not have access to "all health facilities" in their cities and 47% did not have "all general facilities" and 39% did not have "all food facilities". The first two categories were less restrictive and may have gathered more heterogeneous situations.

Regarding barriers in the close vicinity, living in a neighbourhood with poor-quality pedestrian areas and lack of places to rest increased the likelihood of having OADL difficulties, as found in other studies [12, 18, 20, 36, 74, 75]. We further evidenced a clear gradient in pressure on individuals with increasing numbers of FLs. Interestingly, up to 6 FLs, individuals with these barriers were more likely to report difficulties without resorting to assistance, with decreasing pressure. After this threshold, although the barriers increased the probability of having difficulties, the distinction between being assisted and coping with difficulties without assistance was not significant. Unlike other studies, we did not observe any significant associations with environmental barriers such as the presence of hills and slopes [17] or the absence/presence of public toilets [76]. Finally, regarding the immediate environment, using stairs/steps to get out lowered the probability of having no OADL restrictions, as found in several studies [12, 14], with increasing pressure with the number of FLs. Up to 3 FLs, individuals were more likely to resort to assistance; above this threshold, we found no significant distinction for being assisted or not. Our results suggest that improving the environment around homes and neighbourhoods could contribute to reducing risks of disability for all older people whatever their functional status. But we did not observe any significant differences on whether individuals used assistance to perform outdoor daily activities, especially when functional status had deteriorated further.

This research presents several limitations. First, our study, as is the case for most of the studies cited here, used cross-sectional datasets [77]. Therefore, we could not identify the cumulate and short-term effects of barriers [4], neither could we disentangle causal and selection effects. The environment could have acted either as a protection or as pressure on individuals. This association could also result from a composition effect: individuals with better socioeconomic resources tend to settle in areas endowed with amenities close by and in safe neighbourhoods, and they are more likely to be healthy and independent [78]. The association could also result from a selection effect, whereby the most independent people tend to move to (or stay in) areas with amenities suited to their needs. Second, the measures of OADL difficulties and environmental barriers in the nearby environment were self-reported and could reinforce one another. Previous studies have shown that individuals with mobility limitations report more environmental barriers in their environment than their counterparts without such limitations [79, 80]. Moreover, individuals who receive assistance could underestimate obstacles. This could explain the fact that these physical barriers did not show any association with the resort to assistance, while this could be expected. Even if some studies have recommended using such measures to integrate individuals’ perception as part of their actual context [14, 79, 80], we recognise that the measures could have impacted our conclusions. This was a reason for using several approaches to the barriers with different collection modes, to pinpoint potential methodological issues. Third, the data on facilities, based on driving distance between city hall and facilities, was not optimal as it did not reflect the considerable neighbourhood-level differences within the cities [52, 53]. A finer measure of the walking distances between homes and facilities could yield more precise results, but this city-level approach has already provided convincing data. Fourth, other environmental features have been identified in the literature, but could not be taken into consideration here, such as noisy road traffic [16], access to public transport [12, 81], the aesthetics of the neighbourhood [82], the presence of green spaces and water [13] and more broadly, high-quality and safe walking areas [75]. Also, we did not have information on how rural or urban the area of residence was, nor on the socioeconomic and social context of the city, which could have helped to adjust our model [78]. Unaccounted-for interactions between environmental features (for example, the association between OADL restrictions and good accessibility of food outlets may be stronger in walkable neighbourhoods) and between environmental features and other unmeasured neighbourhood characteristics (such as social cohesion or residential segregation) could also be considered. Fifthly, we did not have information on the nature and level of severity of mental illness or substance use disorders, which could play a role influencing decisions on walking out and shopping, and ultimately on independence, independently from functional status and the neighbourhood physical environment. Finally, in our analyses, we did not have the opportunity to consider racial/ethnic minorities, who can interact differently with environment [83, 84].

Our study highlighted interactions between functional status and environmental barriers in relation to disability which need to be confirmed by future studies in other contexts. To explore the issue further, more systematic and refined measures of the physical, socioeconomic and social context in surveys could help provide a clearer idea of the environmental settings that preserve independence. Further studies could explore how functional status interacts with environmental barriers according to gender, socio-economic resources, and racial/ethnic group.

## 5. Conclusion

One major contribution of this study was that is has shown that environmental barriers significantly interact with FLs, restricting individuals in OADL. We found consistent evidence for a contribution of environmental barriers to OADL restrictions, whatever the barrier. We found growing pressure from the environment on OADL difficulties according to the number of limitations; unlike previous studies, we did not find a threshold above which barriers mattered. Neighbourhoods endowed with high diversity of food outlets are important for OADL activities from the very early stages of functional decline and all along the gradient. However, we found different conclusions depending on whether or not they favoured the resort to assistance. This was only clearly the case for food outlet diversity, which appeared to preserve functional independence and reduce need for assistance. However, we found that the environmental influence on resort to assistance decreased as we expected, with the severity of the status. In surveys, more refined measures of barriers and further investigations applying an intersectional approach could help provide a clearer idea of the dynamic processes through which neighbourhood contexts and individual characteristics interact to determine functional independence at older ages.

## Supporting information

S1 Table Distribution of difficulties and resort to assistance for each outdoor activity and the summary measure. CARE-Seniors Ménages Survey (60+ with at least 1 FL), 2015, France.
(PDF)

S2 Table Questions and items selected to assess the functional status of the participants. CARE-Seniors Ménages Survey (60+ with at least 1 FL), 2015, France.
(PDF)

S3 Table Relative Adjusted Predictions at Representative values (APRs) of OADL difficulties and resort to assistance, for 1 to 10+ severe FLs (Calculations based on APRs estimated in model 2). CARE-Seniors Ménages Survey (60+ with at least 1 FL) 2015, France.
(XLSX)
