# Supplementary material for: Environmental barriers matter from the early stages of functional decline among older adults in France
Source: PLoS One. 2022 Jun 22;17(6):e0270258. doi: 10.1371/journal.pone.0270258 (PMC9216542; doi:10.1371/journal.pone.0270258)
Supplement: S1 Table — CARE-Seniors Ménages Survey (60+ with at least 1 FL), 2015, France. (PDF) [file pone.0270258.s001.pdf]

S1 Table. Distribution of difficulties and resort to assistance for each outdoor activity and the summary measure. CARE-Seniors Ménages Survey (60+ with at least 1 FL), 2015, France.

	**No OADL difficulties**<br>N (%)	**Difficulties but no resort to assistance**<br>N (%)	**Resort to assistance**<br>N (%)
Shopping	2,601 (61.5)	870 (9.3)	3,980 (29.2)
Getting out of the house	4,006 (75.9)	1,627 (12.2)	1,818 (11.9)
Using public transports	3,164 (67.5)	1,761 (14.0)	2,526 (18.5)
Carrying out administrative procedures	3,938 (73.0)	617 (5.9)	2,896 (21.1)
Summary measure	1,964 (52.5)	966 (11.6)	4,521 (35.9)

% are weighted
