# Supplementary material for: Environmental barriers matter from the early stages of functional decline among older adults in France
Source: PLoS One. 2022 Jun 22;17(6):e0270258. doi: 10.1371/journal.pone.0270258 (PMC9216542; doi:10.1371/journal.pone.0270258)
Supplement: S2 Table — CARE-Seniors Ménages Survey (60+ with at least 1 FL), 2015, France. (PDF) [file pone.0270258.s002.pdf]

S2 Table. Questions and items selected to assess the functional status of the participants. CARE-Seniors Ménages Survey (60+ with at least 1 FL), 2015, France.

Nature of functional limitation	Question	Different possible answers	Values attributed to the different answers to calculate the number of severe functional limitations
**Sensory**	1. Can you see clearly the printed text of a newspaper with your glasses, or if you wear any, with your contact lenses?	a. Yes, without any difficulty<br>b. Yes, with some difficulty	0
		c. Yes, with a lot of difficulty<br>d. No, you are not at all able	1
	2. Can you see clearly the face of a person, 4 metres away (on the other side of the street), with your glasses, or if you wear any, with your contact lenses?	a. Yes, without any difficulty<br>b. Yes, with some difficulty	0
		c. Yes with a lot of difficulty<br>d. No, you are not at all able	1
	3. Can you hear a conversation with another person in a silent room with your hearing aid if you have one?	a. Yes, without any difficulty<br>b. Yes, with some difficulty	0
		c. Yes, with a lot of difficulty<br>d. No, you are not at all able	1
	4. Can you hear what is said in a conversation with several people with your hearing aid if you have one?	a. Yes, without any difficulty<br>b. Yes, with some difficulty	0
		c. Yes with a lot of difficulty	1

		d. No, you are not at all able	
**Motor**	5. Can you walk 500 metres on flat land without the help of anyone, without a walking stick, or a crutch or a Zimmer frame?	a. Yes, without any difficulty<br>b. Yes, with some difficulty	0
		c. Yes, with a lot of difficulty<br>d. No, you are not at all able	1
	6. Can you climb up or down some stairs without the help of anyone, without a walking stick or a handrail?	a. Yes, with difficulty<br>b. Yes, with some difficulty	0
		c. Yes, with a lot of difficulty<br>d. No, you are not at all able	1
	7. Can you lower yourself or kneel without the help of anyone, or an object (walking stick, table, chair…)?	a. Yes, without any difficulty<br>b. Yes, with some difficulty	0
		c. Yes, with a lot of difficulty<br>d. No, you are not at all able	1
	8. Can you carry a shopping bag of 5 kilos for a distance of 10 metres without the help of anyone, or a Zimmer frame, a walking stick or another walking aid?	a. Yes, without any difficulty<br>b. Yes, with some difficulty	0
		c. Yes, with a lot of difficulty<br>d. No, you are not at all able	1
	9. Can you raise your arms (to reach an object for instance)?	a. Yes, without any difficulty<br>b. Yes, with some difficulty	0
		c. Yes, with a lot of difficulty<br>d. No, you are not at all able	1
	10. Can you use your hands and your fingers (to open a door, handle a water tap, grasp a pen, use scissors… for instance)?	a. Yes, without any difficulty<br>b. Yes, with some difficulty	0
		c. Yes, with a lot of difficulty<br>d. No, you are not at all able	1
		a. Yes, without any difficulty	0

	11. Are you able to check your stools and urine?	b. Yes, with some difficulty	
		c. Yes, with a lot of difficulty<br>d. No, you are not at all able	1
**Cognitive**	12. Do you sometimes not remember what time of the day it is?	a. No<br>b. Yes, sometimes	0
		c. Yes, often	1
	13. In the course of the day, do you sometimes have memory blanks?	a. No<br>b. Yes, sometimes	0
		c. yes, often	1
	14. Do you feel that your memory does not work as well as the memory of people your age?	a. No<br>b. Yes, somtimes	0
		c. Yes, often	1
	15. Have you felt a deterioration of your memory in the last 6 months?	a. No<br>b. Yes, sometimes	0
		c. Yes, often	1
	16. Do you have difficulty concentrating for more than 10 minutes?	a. No<br>b. Yes, sometimes	0
		c. Yes, often	1
	17. Do you have any difficulty solving daily-living problems (such as finding your way or counting up money)?	a. No<br>b. Yes, sometimes	0
		c. Yes, often	1
	18. Do you have any difficulty understanding others or getting others to understand you?	a. No<br>b. Yes, sometimes	0
		c. Yes, often	1
		1. No, no difficulty at all<br>2. Yes, with some difficulty	0

	19. In your day-to-day activities, do you find it difficult to establish social links with other people?	3. yes, with a lot of difficulty	1
